# Cerebellar Glioblastoma: A Literature Review and Case Analysis

**DOI:** 10.7759/cureus.55135

**Published:** 2024-02-28

**Authors:** Ivan Tarev, Asen Cekov

**Affiliations:** 1 Department of Neurosurgery, Acibadem City Clinic Tokuda Hospital, Sofia, BGR

**Keywords:** small-cell glioblastoma, survival, chemotherapy, radiotherapy, surgery, posterior cranial fossa

## Abstract

Glioblastoma multiforme is one of the most common primary intracranial tumors with a particularly aggressive behavior. It usually develops in the cerebral hemispheres, with infratentorial localization being extremely rare. If located in the posterior cranial fossa, glioblastoma most often presents with symptoms of increased intracranial pressure and impaired cerebellar function. In this article, we present a case of small-cell glioblastoma, which is a rare histological variant of this type of high-grade glioma, situated in the cerebellum. A 31-year-old woman was admitted to the neurosurgery department with severe headache, impaired balance, and weakness in the right arm. Magnetic resonance imaging of the brain showed evidence of a lesion with solid and cystic components in the right cerebellar hemisphere. The latter was surgically removed and the histological examination determined the diagnosis of cerebellar small-cell glioblastoma. The treatment of this patient included a combined approach, i.e., radiotherapy and chemotherapy with temozolomide after surgery. Follow-up for a period of more than two years was done and the patient showed no significant clinical symptoms. There was no evidence of recurrence on follow-up imaging studies.

## Introduction

Glioblastoma is one of the most common primary malignant tumors of the central nervous system, accounting for about 50% of all primary intracranial tumors [1]. It usually affects patients in their fifth or sixth decade of life [2]. It originates simultaneously from the glial cells of the brain, growing infiltratively and spreading diffusely in the direction of white matter fibers. Primary cerebellar glioblastoma accounts for about 1% of all glioblastomas according to most studies [2,3], with the small-cell variant making the case even rarer [4]. Some studies report a poorer prognosis in patients with primary infratentorial glioblastomas, with a median survival of three to seven months [5], while others have reported a similar survival to supratentorial glioblastomas [6]. The mean age of onset of infratentorial glioblastoma is 50.3 years [7,8], which is lower than that of supratentorial glioblastoma. In addition, cerebellar glioblastoma is extremely rare in the elderly [9]. It is characterized by symptoms of increased intracranial pressure and cerebellar dysfunction.

## Case presentation

**Figure 1:**
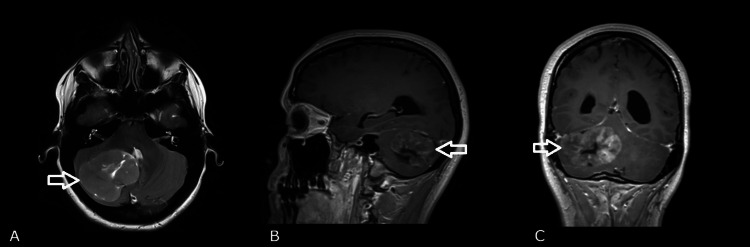
Preoperative magnetic resonance imaging showing a lesion (white arrow) in the right cerebellar hemisphere with axial dimensions of 51 x 48 mm (A: axial; B: sagittal; C: coronal).

A 31-year-old woman was admitted to the neurosurgery clinic due to severe headache and neck pain persisting for the past month, accompanied by impaired balance and gait instability which emerged in the last week. Periodic episodes of vertigo and weakness in the right arm were reported. The neurological examination showed moderately lively reflexes, more pronounced on the right, Babinski /+/ on the right, and the presence of symptoms of cerebellar dysfunction, namely, ataxia, dysmetria, and dysdiadochokinesia. Magnetic resonance imaging (MRI) revealed the presence of a well-defined polylobulated formation in the right cerebellar hemisphere, with axial dimensions of 51 × 48 mm (Figure 1). The lesion consisted of a solid component with hypointense characteristics compared to the cerebral white matter on T1-weighted imaging sequences and of higher signal on T2-weighted imaging sequences, along with a fluid component of high signal on T2-weighted imaging located between the solid components. After administration of intravenous (IV) contrast material, the solid part of the formation increased its heterointensity. The finding had a significant mass effect on the surrounding structures: it displaced the midline up to 15 mm contralaterally; compressed the IV ventricle, causing moderate obstructive hydrocephalus; compressed the adjacent brainstem at the level of the pons and mesencephalon; and caused initial wedging of the cerebellar tonsils.

**Figure 2:**
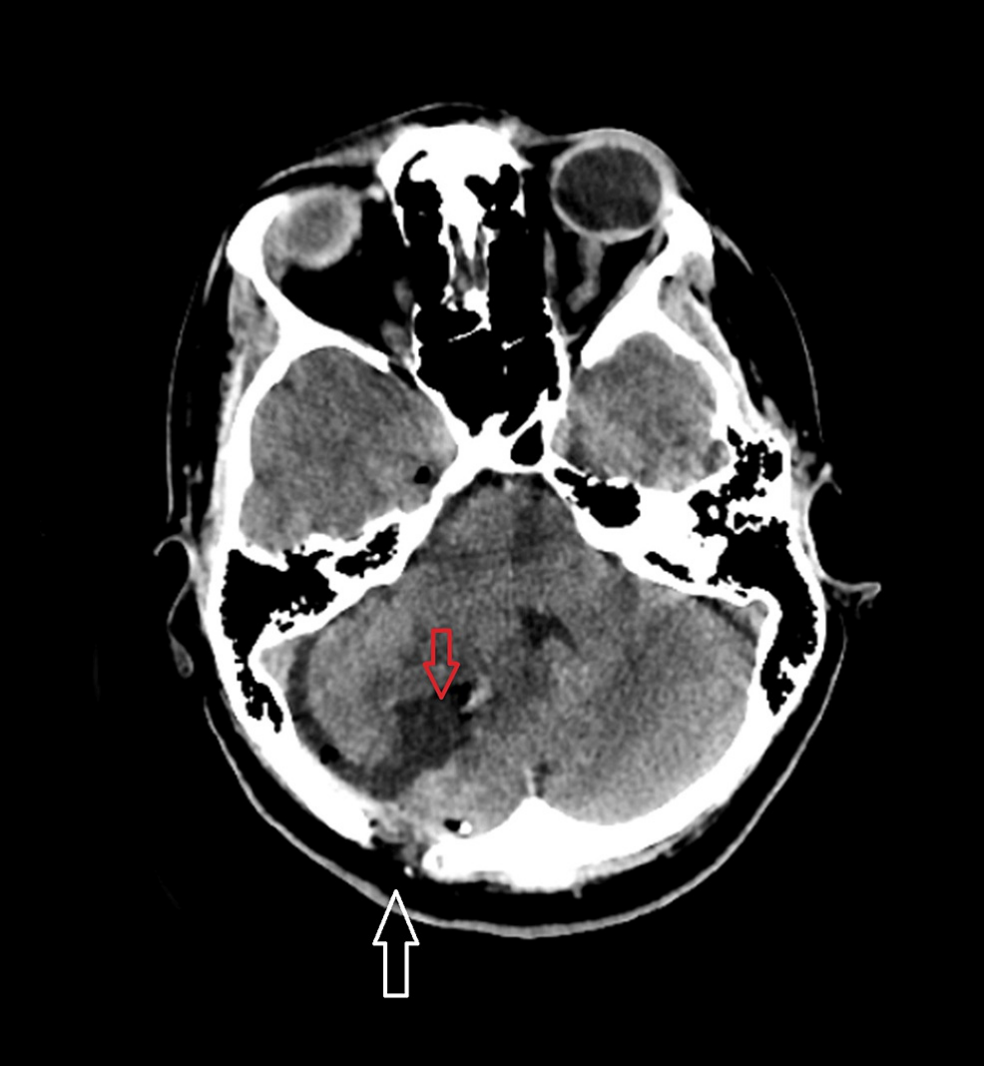
Postoperative computed tomography scan a day after the surgery showing a part of the craniectomy (white arrow) and the area of a visibly totally extirpated tumor (red arrow).

The operative intervention was performed with the patient in the left lateral position on the operating table. A paramedian suboccipital skin incision was made and a right suboccipital craniectomy was performed. Intraoperatively, using ultrasound, we found a profusely bleeding tumor with indistinct borders to the surrounding normal brain tissue. The latter was composed of solid and cystic components and underwent radical resection with part of the surrounding cerebellar parenchyma. Material for histological verification was preserved. On the first postoperative day, a computed tomography (CT) scan of the brain was performed, which showed total extirpation of the lesion and the presence of minimal gas inclusions and hemorrhagic foci in the area of the previous tumor (Figure 2). The patient experienced a smooth postoperative period, without complications, and was discharged on the fifth postoperative day with significant improvement in neurological symptoms.

**Figure 3:**
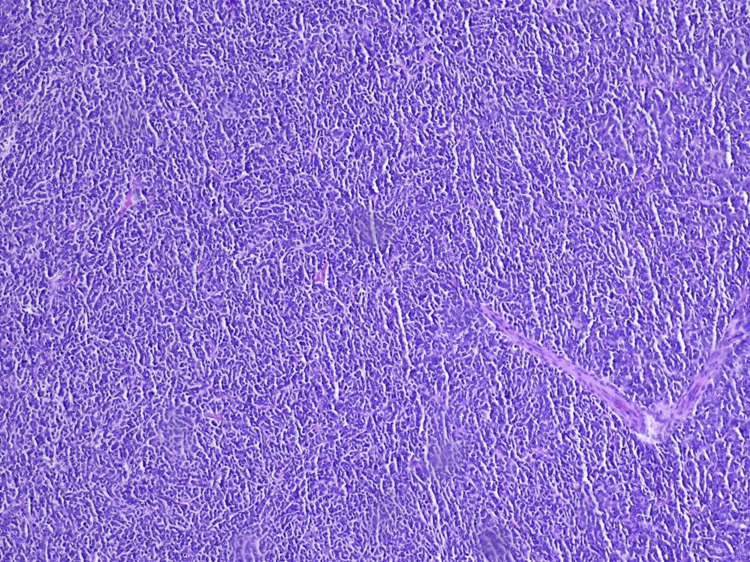
Highly monomorphic cell population characterized by small, round to slightly elongated, densely packed cells with mildly hyperchromatic nuclei, high nuclear:cytoplasmic ratio, and modest atypia (hematoxylin and eosin staining).

**Figure 4:**
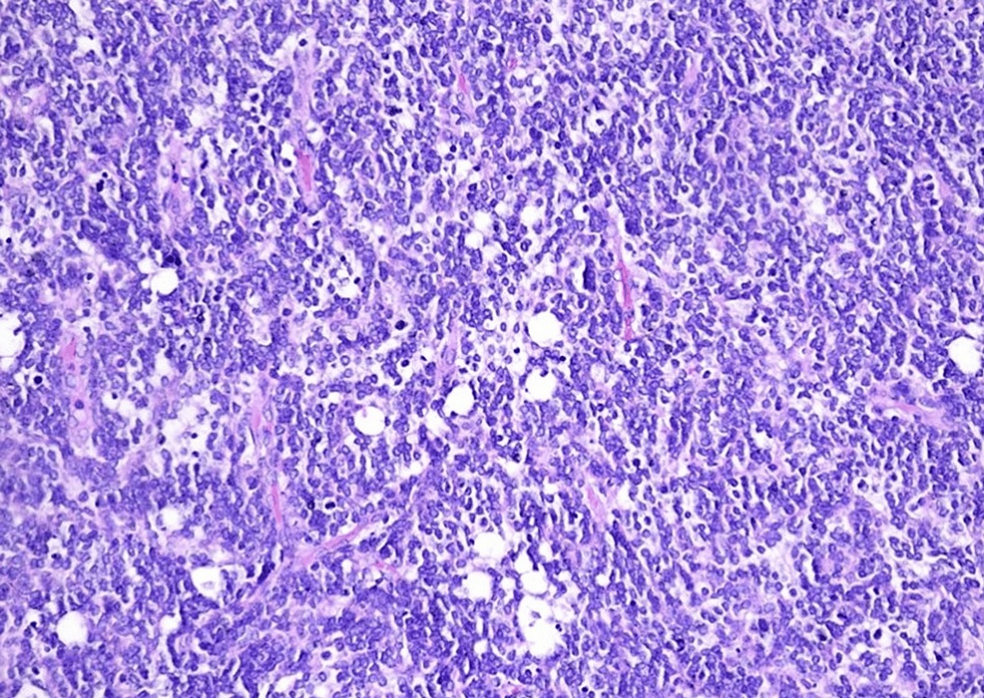
Highly monomorphic cell population characterized by small, round to slightly elongated, densely packed cells with mildly hyperchromatic nuclei, high nuclear:cytoplasmic ratio, and modest atypia (hematoxylin and eosin staining).

**Figure 5:**
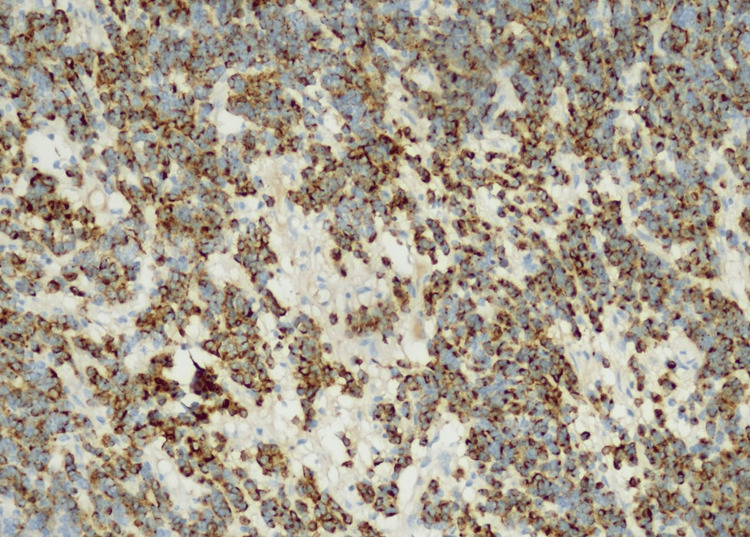
Glial fibrillary acidic protein immunoreactivity highlights delicate processes.

**Figure 6:**
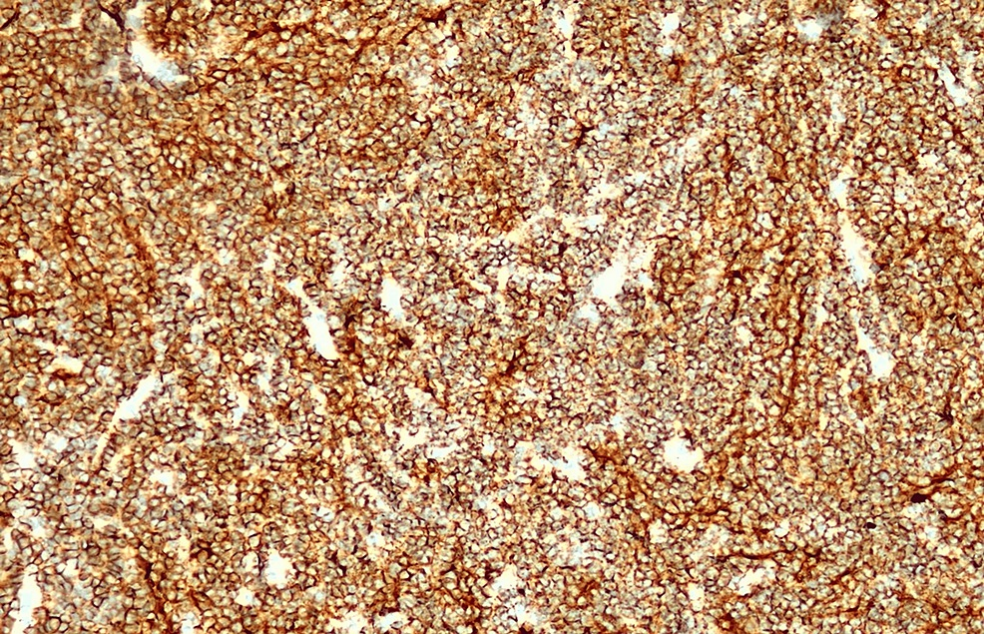
CD56 immunoreactivity.

**Figure 7:**
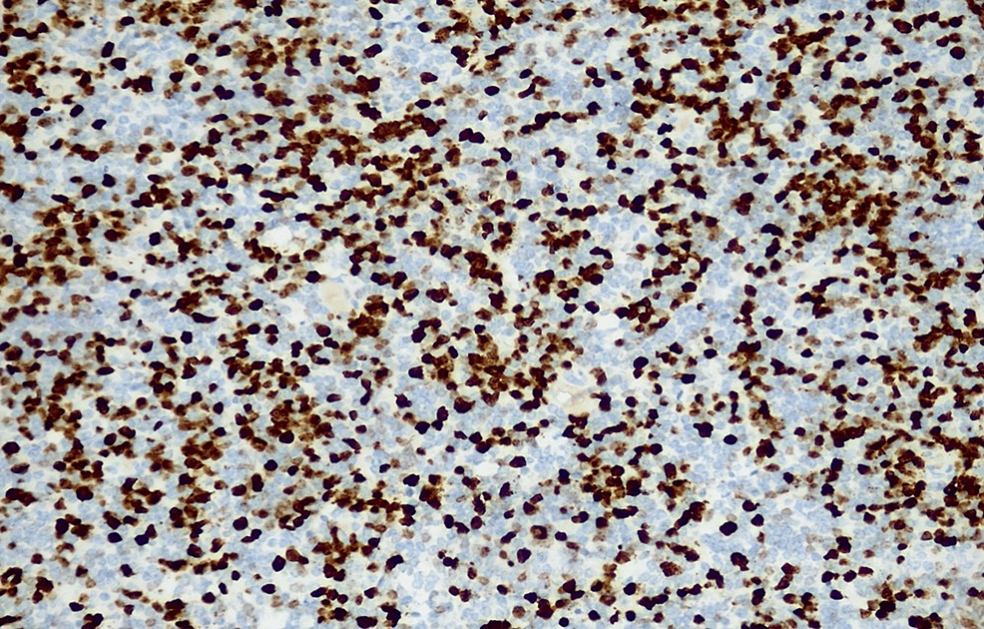
Ki67 demonstrates a high proliferation index of about 70% of tumor cells.

The histological examination showed massive infiltration of a malignant, hypercellular tumor, composed of monomorphic, small, round cells, with scant cytoplasm and large, atypical, hyperchromic nuclei, showing modest atypia (Figures 3, 4). Immunohistochemistry showed a slightly positive glial fibrillary acidic protein (GFAP) reaction in a large proportion of tumor cells and a weakly intense, positive synaptophysin reaction in a smaller proportion (Figures 5, 6). Ki67 demonstrated a high proliferation index of about 70% of tumor cells (Figure 7). CD56 showed a positive reaction. The diagnosis of small-cell glioblastoma in the posterior cranial fossa was established.

After the histological confirmation of the diagnosis, the patient’s treatment proceeded with radiotherapy and chemotherapy with temozolomide. The course of combined radiation-chemotherapy was started 32 days after surgery and the patient received a total dose of 54 Gray along with 75 mg/m^2^ temozolomide daily. Initially, the craniospinal axis and central nervous were irradiated in 17 fractions of 1.8 Gray for a total of 30.6 Gray. A boost dose was then administered to the cerebellum in 13 fractions of 1.8 Gray for a total of 23.4 Gray. The patient showed good tolerance to radiation exposure and did not show symptoms of an acute radiation reaction.

The treatment regimen proceeded with six cycles of chemotherapy, comprising five days of temozolomide at a dosage of 150 mg/m^2^, followed by a hiatus on the 28th day before repeating the cycle. The dosage was not increased to 200 mg/m^2^ due to challenging tolerance and the onset of borderline thrombocytopenia.

**Figure 8:**
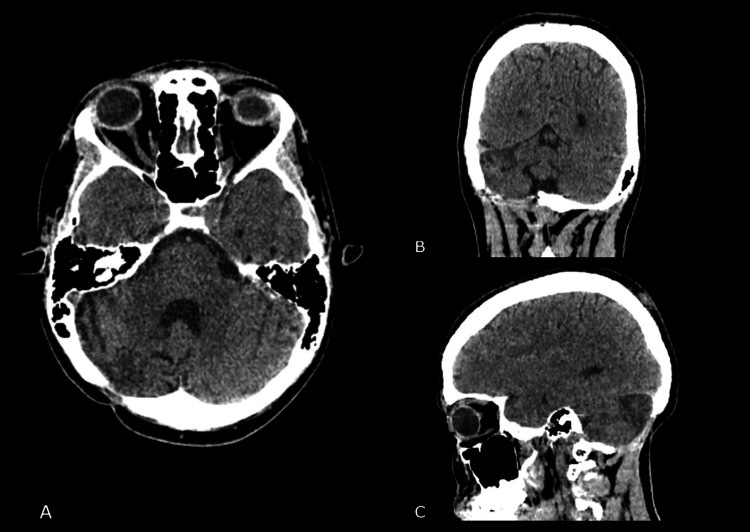
Follow-up computed tomography scan one year after surgery (A: axial; B: coronal; C: sagittal).

**Figure 9:**
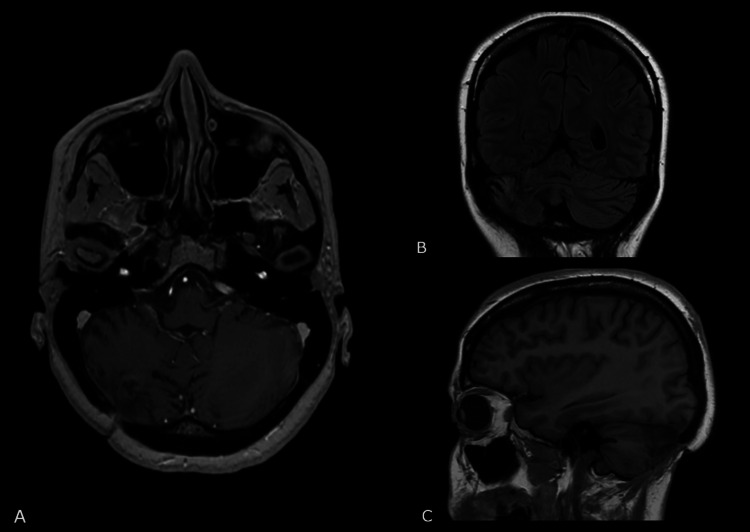
Follow-up magnetic resonance imaging two years after surgery (A: axial; B: coronal; C: sagittal).

On regular follow-ups of the patient’s condition at check-ups for more than two years, the patient had no significant clinical signs and no neurological symptoms, preserving a normal and active lifestyle. We conducted follow-up MRI and CT examinations every three to four months following the intervention, revealing no signs of recurrence of the lesion (Figures 8, 9).

## Discussion

Glioblastoma is one of the most common primary malignant intracranial tumors. It occurs in people of different ages, but the peak incidence is in the sixth decade of life [1,10]. Its infratentorial localization is extremely rare, accounting for fewer than 1% of all glioblastomas [1,2]. Usually, the clinical picture is characterized by symptoms of increased intracranial pressure, such as headache, nausea, and vomiting. Patients often report vertigo, impaired balance, and neck pain [11,12]. Quantitative changes in consciousness are possible ranging from obnubilation to a comatose state with the development of severe acute hydrocephalus.

The diagnosis of cerebellar glioblastoma is rarely assumed preoperatively, although there are certain radiographic features suggesting it. The most common suspicions in terms of differential diagnosis are metastatic tumors and anaplastic astrocytoma, as well as medulloblastoma in younger people. On contrast-enhanced CT, glioblastoma usually appears as a solid tumor with central hypodense areas suggestive of necrosis. Peritumoral edema in glioblastomas is less than that in cerebellar metastases [10]. On MRI, cerebellar glioblastoma presents as a heterointense mass composed of solid and cystic components. Choline levels on MRI spectroscopy in the perilesional areas are high in glial tumors [13]. Relative cerebral blood volumes in the peritumoral regions, calculated by perfusion-weighted MRI, are significantly higher in gliomas than in metastatic lesions [2]. Cerebellar abscess and infarction should also be excluded. On CT scan, cerebellar abscess appears hypodense with surrounding edema and contrast ring enhancement, while infarction is hyperdense in acute stages and then becomes hypodense in subacute/chronic stages.

Small-cell glioblastoma is a rare histopathological variant of the classic glioblastoma multiforme. According to literature data, it represents about 11% of the total number of glioblastomas [3,14]. It is composed of small, round neoplastic cells with minimal atypicality and high cell density, exhibiting high mitotic activity and nuclear/cytoplasmic ratio. Radiologically, it does not differ significantly from classic glioblastoma. Multifocality in this variant is more common. It is possible to have varying degrees of necrosis and microvascular proliferation, and these features are not essential for the diagnosis as in classic glioblastoma multiforme. The definitive diagnosis of small-cell glioblastoma is provided by histopathological examination, in addition to which it may be necessary to perform an immunohistochemical examination to exclude other differential diagnoses.

Immunohistochemical staining patterns may detect positivity for GFAP, a marker indicating astrocytic differentiation, although to a lesser degree compared to conventional glioblastoma. Positive staining for synaptophysin is often observed, indicating neuroendocrine differentiation, a characteristic feature of small-cell glioblastoma. Positive neuron-specific enolase staining further supports the neuroendocrine differentiation of tumor cells in small-cell glioblastoma. Small-cell glioblastoma typically exhibits high Ki-67/MIB-1 staining due to its elevated proliferative activity, aiding in tumor grading and predicting aggressiveness. Aberrant P53 staining patterns may indicate p53 gene mutations, commonly seen in glioblastomas.

In terms of molecular alterations, classical glioblastomas often harbor mutations in genes such as the epidermal growth factor receptor and phosphatase and tensin homolog. However, small-cell glioblastoma may present a distinct mutational landscape. Small-cell glioblastoma is typically categorized as IDH-wildtype, indicating the absence of mutations in isocitrate dehydrogenase 1 or 2 genes. Conversely, IDH mutations, commonly found in lower-grade gliomas and some classical glioblastomas, are infrequently observed in small-cell glioblastoma.

Considering the malignant nature of high-grade glial tumors, the gold standard in surgical treatment is to perform a large total resection of the formation, which often, due to infiltrative span and growth, is not feasible. Suboccipital craniectomy is the most common surgical procedure used to approach these tumors. Radiotherapy is a well-established adjuvant method of treatment [15]. Some studies recommend local irradiation of limited fields involving the posterior cranial fossa, the brainstem, and the upper cervical segment of the myelon [16]. Others support the thesis of postoperative irradiation of the entire brain and craniospinal axis [5]. Temozolomide chemotherapy after surgical resection has been shown to improve overall survival.

Cerebellar and supratentorial glioblastoma have similar biological behavior [17]. Overall survival rates vary strongly according to different publications and reports. Median survival is significantly shorter in elderly patients compared to younger ones [18]. According to Weber et al., median survival is about 10 months [8]. The presence of brainstem infiltration by the tumor is a poor prognostic factor.

## Conclusions

As primary infratentorial glioblastoma is rare, its pathogenesis is still not well understood. The small-cell variant makes the case even rarer. Definitive diagnosis is determined by taking a biopsy and histological examination. Its treatment consists of surgical resection, if possible total, followed by radiotherapy and chemotherapy. An important prognostic factor in cerebellar glioblastoma is the presence of brainstem infiltration. Despite the radicality of the treatment applied, the average survival duration is short.
